# A Modified Phase Cycling Method for Complex-Valued MRI Reconstruction

**DOI:** 10.1155/2020/8846220

**Published:** 2020-11-18

**Authors:** Wei He, Yu Zhang, Junling Ding, Linman Zhao

**Affiliations:** ^1^Department of Computer Science and Technology, Xinyang Normal University, Xinyang 464000, China; ^2^Henan University of Chinese Medicine, Zhengzhou 450046, China

## Abstract

The phase cycling method is a state-of-the-art method to reconstruct complex-valued MR image. However, when it follows practical two-dimensional (2D) subsampling Cartesian acquisition which is only enforcing random sampling in the phase-encoding direction, a number of artifacts in magnitude appear. A modified approach is proposed to remove these artifacts under practical MRI subsampling, by adding one-dimensional total variation (TV) regularization into the phase cycling method to “pre-process” the magnitude component before its update. Furthermore, an operation used in SFISTA is employed to update the magnitude and phase images for better solutions. The results of the experiments show the ability of the proposed method to eliminate the ring artifacts and improve the magnitude reconstruction.

## 1. Introduction

In traditional magnetic resonance imaging (MRI), reconstructing an image requires the full Fourier domain (*k*-space) samples which are complex values and usually on a regular Cartesian grid. The full acquisition leads to an extremely time-consuming scan process. Compressed sensing (CS) has proven to be effective to reconstruct high-quality images from the incomplete set of *k*-space samples, accelerating the scan process [1–5]. And many researchers combine CS and parallel imaging techniques (CS-PI) to further speed up the MRI acquisition [6–8]. Most approaches applying CS in MRI reconstruction [2–8] focus on the magnitude recovery, leaving the phase reconstruction less studied. However, MRI images are complex-valued, including magnitude and phase parts. And the phase of MRI signal also includes important information, such as field inhomogeneity or the velocity of blood flow [9], and can be utilized to assess *B*_0_ field inhomogeneity and obtain clinically relevant physiological parameters [10]. Therefore, the phase map is of interest in many applications such as field map estimation [11] and phase-contrast imaging [12, 13]. If we can reconstruct accurate complex-valued MR image, i.e., its magnitude and phase maps simultaneously, from under-sampled raw *k*-space data, it will make CS more generally applicable in MRI.

Zibetti and De Pierro [14] proposed a new penalty to separate the regularization of magnitude and phase, enforcing the magnitude to be sparse in finite differences domain and the phase to be smooth in first-order roughness. This algorithm gives some better results than standard CS. But it is only applicable for first-order differences operators in CS regularization, which is not the optimal choice. Moreover, in this method, the phase is still not reconstructed independently from the magnitude, because the parameter for the phase regularization term is constrained by the corresponding magnitude. It causes low SNR of the phase image in the locations having low magnitude. At last, the initialization for phase should be good when the first-order phase difference is large, as the regularization term for phase is not convex here.

Some new regularization terms for phase component, exploiting the periodicity of phase, were introduced by Zhao et al. [15] to improve phase results. In this method, discontinuities are allowed to exist in phase, since the smoothness penalty for phase is modified. However, it has more computational complexity, e.g., about ten times, than the conventional CS.

The phase cycling method [16] proposed by Ong et al. is a state-of-the-art algorithm for complex-valued MR image recovery. However, this approach follows the variable density Poisson-disk plus partial Fourier (PDPF for short in this paper) subsampling scheme that is impractical for MRI hardware in 2D acquisition [17]. When random sampling is only imposed in the phase-encoding direction [18] (RSPe for short in this paper) on a 2D Cartesian grid, which is workable in practical MRI 2D acquisition, there are usually some noticeable artifacts in magnitude image, especially of brain data. The artifacts will be shown in Section 2.

In this paper, we utilize the smoothing property of total variation (TV) norm to remove the artifacts before each magnitude update under some sampling scheme and apply an update operation in the smoothing-based FISTA (SFISTA) [19] to improve the image recovery. The detailed procedure of this modified phase cycling method will be given in Section 2.

## 2. Materials and Methods

The cost function of the phase cycling method is

(1) $$\begin{array}{c} \frac{1}{2}{\left\|y-A\left(m∙e^{ip}\right)\right\|}_{2}^{2}+g_{m}\left(m\right)+g_{p}\left(p\right), \end{array}$$

where *m* and *p* are the magnitude and phase images, respectively, *y* denotes the subsampled *k*-space measurements, **A** is the system matrix for the complex-valued images, ∙ denotes the element-wise multiplication, *e*^*ip*^ is the element-wise exponential operator of the matrix *ip*, and *g*_*m*_(*m*) and *g*_*p*_(*p*) are the regularization functions for *m* and *p*, respectively. The first term in Equation (1) indicates the data fidelity, where **A** is normally the product of the Fourier sampling operator and the sensitivity operator for multicoil data and reduces to a subsampling Fourier matrix for single-coil data. For convenience, we call this data fidelity term *f*(*m*, *p*).

The alternating minimization is performed to update the magnitude and phase images separately in this algorithm. And the proximal method [20, 21] is applied to solve each subproblem in the phase cycling method. The highlight of this method is that it shifts the phase wraps randomly over iterations to avoid significant error accumulating at the same location. It is because the phase wraps are artifacts in the initial solution and penalized by phase regularization in each iteration, resulting in errors to accumulate at the same position over iterations [16]. In this way, the magnitude and phase images are updated at iteration *k* as follows:

(2) $$\begin{array}{c} mg=m_{k}-\rho_{m}\nabla_{m}f\left(m_{k},p\right), \end{array}$$

(3) $$\begin{array}{c} m_{k+1}={\text{prox}}_{\rho_{m}}\left(g_{m}\left(m\right)\right)\left(mg\right), \end{array}$$

(4) $$\begin{array}{c} pg=p_{k}-\rho_{p}\nabla_{p}f\left(m,p_{k}\right), \end{array}$$

(5) $$\begin{array}{c} p_{k+1}={\text{prox}}_{\rho_{p}}\left(g_{p}\left(p\right)\right)\left(pg+w_{k}\right)-w_{k}, \end{array}$$

where

(6) $$\begin{array}{c} {\text{prox}}_{\rho }\left(g\right)\left(x\right)∶={\text{argmin}}_{u}\left\{g\left(u\right)+\frac{1}{2\rho }{\left\|u-x\right\|}^{2}\right\}, \end{array}$$

is the proximal operator for function *g*, *ρ*_*m*_ and *ρ*_*p*_ denote the step-size for magnitude *m* and phase *p*, *w*_*k*_ is a phase wrap picked randomly from the set of phase wraps *ω* generated by the initial solution with equal probability.

**Figure 1:**
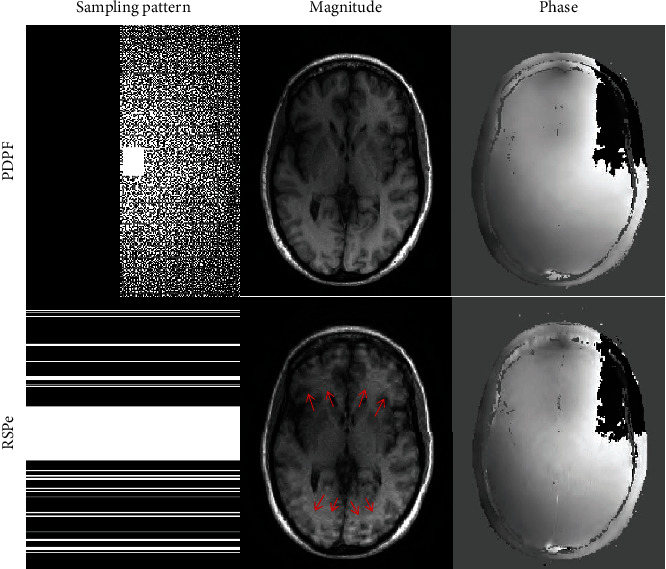
The results of the phase cycling method under two undersampling schemes at 30% sampling rate on a brain dataset.

The phase cycling algorithm performs well based on PDPF undersampling scheme on a 2D Cartesian grid. Nevertheless, when it follows a practical RSPe subsampling scheme in 2D Cartesian acquisition, some artifacts pointed by the red arrows sometimes appear in magnitude image as shown in Figure 1. In addition, as the center of *k*-space data consists of low frequency signals, the central area is fully-sampled in both undersampling schemes to improve the reconstruction quality and compute coil sensitivities for multicoil data.

**Figure 2:**
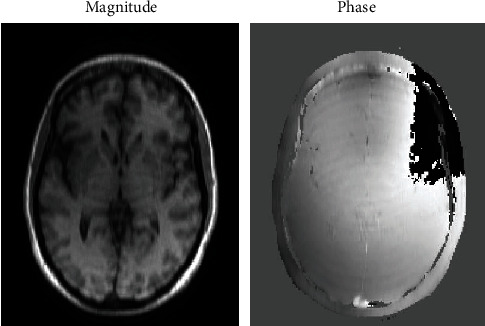
The magnitude and phase images in the initial step under the RSPe sampling pattern at 30% sampling rate on the brain dataset of Figure 1. Both images have artifacts.

Under the RSPe sampling pattern, the collected *k*-space data lack the whole echoes along several phase-encoding lines. Via inverse Fourier transform, the data on these missing lines are zero-padded, generating significant artifacts in the initial step in both magnitude and phase maps (see Figure 2). The artifacts are like arcs generally in the horizontal direction orthogonal to the phase-encoding coordinates. So far, our observation is that, under the RSPe sampling pattern, artifacts tend to happen in the initial step when the magnitude image has some very big values forming lines, arcs, or circles, which is common within brain images.

Since the phase cycling approach effectively averages the artifacts of phase wraps spatially in phase image over iterations by virtue of shifting the phase wraps randomly, the artifacts caused by the RSPe sampling pattern can be averaged incidentally. This can be deduced by comparing the phase maps under the RSPe sampling pattern in Figures 1 and 2. As phase values interact with magnitude values via calculating the gradient of the data fidelity term *f*(*m*, *p*) (see Equations (2) and (4)), the artifacts of magnitude reduce more or less eventually by means of delivering the abovementioned phase image with less or no artifacts to the data fidelity term. However, these errors in magnitude cannot be removed thoroughly in most situations.

In our method, before each update, one-dimensional TV regularization is conducted on the magnitude images to eliminate the artifacts mentioned above. This is inspired by the piecewise-smooth nature of TV norm [22–24]. Since the artifacts appear to be oriented towards the coordinates orthogonal to the phase-encoding direction, the one-dimensional TV regularization of magnitude shown in Equations (7) and (8) is enforced in the phase-encoding direction before each magnitude update in the phase cycling algorithm.

(7) $$\begin{array}{c} {mg}^{\prime }=m_{k}-\rho_{m\text{Tv}}\nabla_{m}f\left(m_{k},p\right), \end{array}$$

(8) $$\begin{array}{c} m_{k\text{TV}}={\text{prox}}_{\rho_{m\text{Tv}}}\left(\gamma {\left\|m\right\|}_{\text{TV}-1\text{D}}\right)\left({mg}^{\prime }\right), \end{array}$$

where ‖*m*‖_TV−1D_ denotes the one-dimensional TV norm in the phase encoding direction, *m*_*k*TV_ denotes the magnitude after the one-dimensional TV regularization for iteration *k*, *ρ*_*m*TV_ is the step-size of the TV regularization, and *γ* is a positive regularization parameter.

Furthermore, inspired by SFISTA [19], the following generalized step called the smoothing-based proximal operator (SPO) in this paper replaces the proximal operator in the phase cycling method:

(9) $$\begin{array}{c} x_{k+1}=\left(1-\frac{\rho }{\mu }\right)x_{k}-\frac{\rho }{\mu }{\text{prox}}_{\rho }\left(\text{g}\left(\text{x}\right)\right)\left(x_{k}\right)+\rho \nabla_{x}f, \end{array}$$

where *ρ* is the step-size, *μ* denotes smooth approximation parameter, and ∇_*x*_*f* represents the gradient of the data fidelity term *f* with respect to the update variable *x*.

The proposed method benefits from the smoothing effect of TV norm and the regularization-solving capacity of SPO.

The pseudocode of the modified phase cycling method is shown as follows:

## 3. Results and Discussion

The proposed approach and the phase cycling method are implemented in MATLAB (MathWorks, Natick, MA) and run on a laptop with a 1.1 GHz Intel Core i7 with 6 multicores, and 16 GB memory. The step-size *ρ*_*m*_ for magnitude update is 1/*λ*_max_(**A**^∗^**A**), and the step-size *ρ*_*p*_ for phase update is 1/(*λ*_max_(**A**^∗^**A**)max(|*m*|^2^)), where *λ*_max_ denotes the maximum eigenvalue calculated analytically. The maximum number of iterations *K* is 100. The *l*_1_ norm on the Daubechies 4 wavelet transform coefficients of magnitude is selected to be function *g*_*m*_(*m*) unless otherwise specified. The smooth approximation parameter *μ* is set as 1 for the best performance of SPO. For each method, the regularization parameters are chosen for its optimal performance in terms of peak-to-signal noise ratio (PSNR) and structural similarity index metric (SSIM) [25].

**Figure 3:**
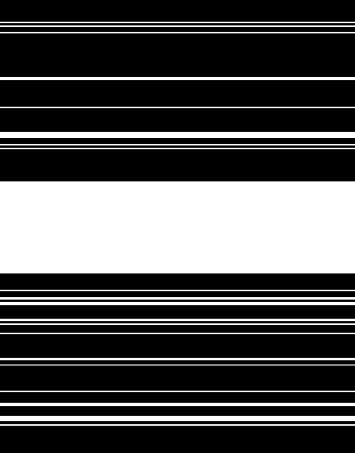
The sampling pattern.

A fully sampled human brain dataset [16], with a 2D image size of 230 × 180 and acquired by an 8-channel head coil, is available in the software package at https://github.com/mikgroup/phase_cycling.git. *l*_1_ regularization is imposed on the Daubechies 4 wavelet domain for the phase image. The positive TV regularization parameter *γ* is set to be 0.005. To get the measurements *y*, data along 20% phase-encoding center lines are fully sampled and the other data are from 10% randomly chosen phase-encoding lines in the rest *k*-space. This undersampling pattern is shown in Figure 3.

**Figure 4:**
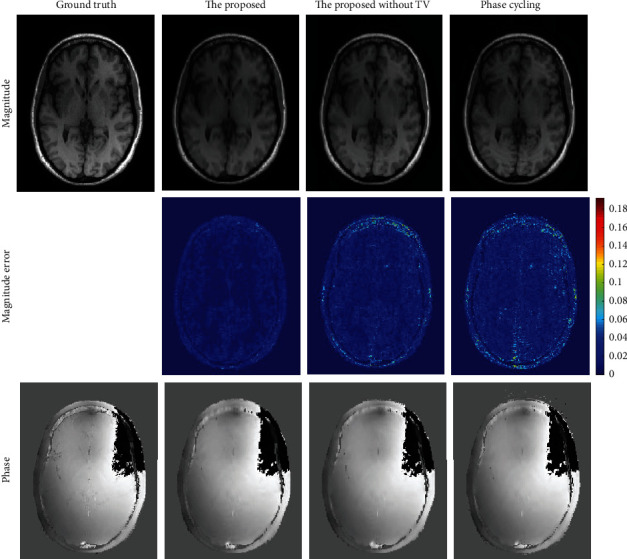
The results on a brain dataset for the proposed algorithm, the proposed method without TV regularization, and the phase cycling method.

Figure 4 shows the results of both methods and the proposed method without TV regularization on the brain dataset. To better display phase results, the background areas of the phase images were roughly masked out by thresholding the bottom 10% of the magnitude images. For the phase cycling algorithm, ring artifacts can be seen in the magnitude image. While, due to SPO, the proposed method without TV regularization reconstructs the magnitude image with less error compared to the phase cycling method, there are still some ring artifacts in the magnitude result. Furthermore, the proposed method removes the ring artifacts and promotes the quality of magnitude recovery significantly owe to the TV regularization and SPO. Comparing the magnitude images and their corresponding error maps of the proposed method with and without 1D TV regularization, it can be deduced that the 1D TV regularization can indeed decrease and even eliminate the ring artifacts. On the other hand, the magnitude image of the phase cycling method has more errors in general, which can be demonstrated by the contrast of the error maps. The three approaches perform comparably with each other in phase reconstruction.

Table 1 lists the PSNRs and SSIMs of the magnitude and phase maps by the proposed and phase cycling methods under the RSPe sampling pattern with a 30% sampling rate. Here, ten distinct RSPe sampling schemes with 30% samples are adopted to verify the robustness of the proposed method. The PSNR of the magnitude images by the proposed method improves about 1~3 dB, while the SSIM promotes about 0.01~0.02.

**Figure 5:**
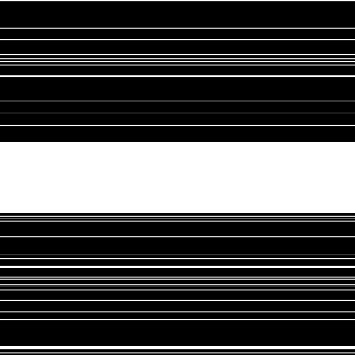
The sampling pattern.

**Figure 6:**
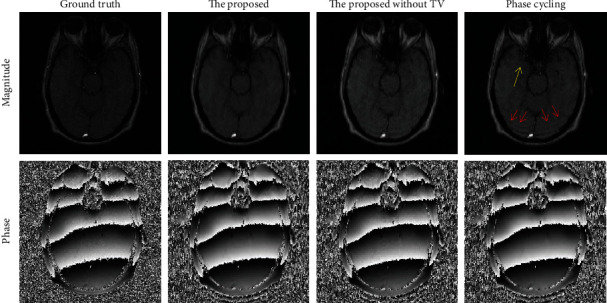
The results on another brain dataset for the proposed algorithm with and without TV regularization and the phase cycling method.

Another 256 × 256 single-coil brain dataset (see Dataset S1 in the Supplementary Material) is used to test the proposed algorithm. *l*_1_ regularization is imposed on the Daubechies 6 wavelet domain for the phase image. *γ* = 0.004. The data in the readout direction are always full sampled. And the random sampling only takes place in phase-encoding direction, with 20% center coordinates and 10% randomly selected coordinates elsewhere. Under this sampling pattern shown in Figure 5, Figure 6 displays the results of the proposed method with and without TV regularization and the phase cycling algorithm. The last method produces several artifacts pointed by arrows in the magnitude result. The artifact pointed by the yellow arrow may be mistaken as tissue in the brain. The proposed algorithm removes these artifacts distinctly thanks to the TV regularization, while without it the proposed method generates some ring artifacts. And the phase images reconstructed by three approaches are nearly the same.

Here, we still do ten experiments under ten different RSPe sampling schemes with 30% samples to calculate the PSNRs and SSIMs of magnitude images by the proposed and phase cycling methods. In terms of PSNR, the former results in 34.9 ± 0.11dB, and the latter results in 33.7 ± 0.15dB. And in terms of SSIM, the former results in 0.91 ± 0.002, and the latter results in 0.903 ± 0.004.

The value of the TV regularization parameter *γ* should be chosen cautiously. When it is too large, the magnitude image will blur and lose much detailed information, whereas too small *γ* renders this artifact-elimination regularization ineffective. In our experience, the value between 0.001 and 0.005 is appropriate.

## 4. Conclusions

We add the one-dimensional TV regularization of magnitude into the phase cycling algorithm, and use SPO to solve the magnitude and phase subproblems. The 1D TV regularization is like a preprocessing step for magnitude in each iteration. And the SPO improves the magnitude recovery to some extent. The modified algorithm can significantly reduce or totally eliminate the artifacts of magnitude by the phase-cycling method under practical RSPe undersampling pattern, while the quality of the reconstructed phase image is comparable to the phase cycling method. However, the performance of the proposed algorithm is sensitive to the value of TV regularization parameter.

## Figures and Tables

**Pseudocode 1:**
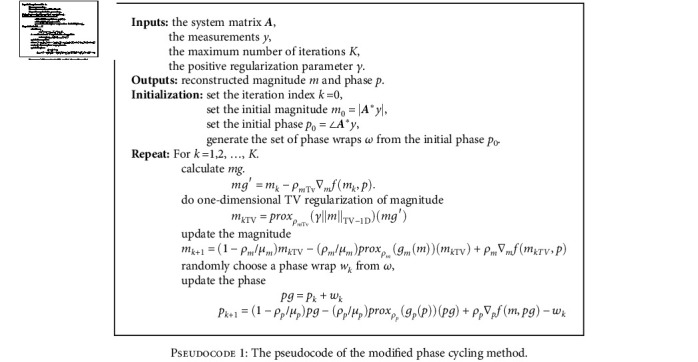


**Table 1:** Comparison of the magnitude results by the proposed and phase cycling methods under RSPe sampling pattern in terms of peak-to-signal noise ratio (PSNR) and structural similarity index metric (SSIM).

Methods	PSNR [dB] (magnitude)	SSIM (magnitude)	PSNR [dB] (phase)	SSIM (phase)
The proposed	38.2 ± 0.45	0.97 ± 0.002	16.9 ± 0.04	0.88 ± 0.002
Phase cycling	36.1 ± 0.81	0.95 ± 0.005	17.0 ± 0.05	0.88 ± 0.004

## Data Availability

The 230 × 180 brain data acquired by an 8-channel head coil are from previously reported studies and datasets, which have been cited. The processed data are available in the software package at https://github.com/mikgroup/phase_cycling.git. The 256 × 256 brain data used to support the findings of this study are included within the supplementary information file “Dataset S1.mat”.

## Supplementary Materials

Supplementary Materials The 256 × 256 brain data are acquired on 1.5 T GE Signa scanner (GE Healthcare, Waukesha, WI) with GRE sequence, supplied in a .mat file named “Dataset S1”.
